# Targeting HDAC with a novel inhibitor effectively reverses paclitaxel resistance in non-small cell lung cancer via multiple mechanisms

**DOI:** 10.1038/cddis.2015.328

**Published:** 2016-01-21

**Authors:** L Wang, H Li, Y Ren, S Zou, W Fang, X Jiang, L Jia, M Li, X Liu, X Yuan, G Chen, J Yang, C Wu

**Affiliations:** 1 Department of Pharmacology, Shenyang Pharmaceutical University, Shenyang, PR China; 2 Benxi Institute of Pharmaceutical Research, Shenyang Pharmaceutical University, Shenyang, PR China; 3 Department of Pathology, Wuhan General Hospital of Guangzhou Command, People's Liberation Army, Wuhan, PR China; 4 Key Laboratory of Structure-Based Drugs Design and Discovery of Ministry of Education, Shenyang Pharmaceutical University, Shenyang, PR China

## Abstract

Chemotherapy paclitaxel yields significant reductions in tumor burden in the majority of advanced non-small cell lung cancer (NSCLC) patients. However, acquired resistance limits its clinical use. Here we demonstrated that the histone deacetylase (HDAC) was activated in paclitaxel-resistant NSCLC cells, and its activation promoted proliferation and tumorigenesis of paclitaxel-resistant NSCLC cells *in vitro* and *in vivo*. By contrast, knockdown of HDAC1, a primary isoform of HDAC, sensitized resistant cells to paclitaxel *in vitro*. Furthermore, we observed that overexpression of HDAC1 was associated with the downregulation of p21, a known HDAC target, in advanced NSCLC patients with paclitaxel treatment, and predicted chemotherapy resistance and bad outcome. In addition, we also identified a novel HDACs inhibitor, SNOH-3, which inhibited HDAC expression and activity, induced cell apoptosis, and suppressed cell migration, invasion and angiogenesis. Notably, co-treatment with SNOH-3 and paclitaxel overcome paclitaxel resistance through inhibiting HDAC activity, leading to the induction of apoptosis and suppression of angiogenesis *in vitro* and in preclinical model. In summary, our data demonstrate a role of HDAC in paclitaxel-resistant NSCLC and provide a promising therapeutic strategy to overcome paclitaxel-acquired resistance.

Non-small cell lung cancer (NSCLC) is one of the main cause of cancer-related death worldwide.^1^ Standard therapeutic approaches for NSCLC include surgical resection, chemo- and radio-therapy.^2^ Due to advanced disease at diagnosis, a majority of patients are not candidates for surgery. Therefore, chemotherapeutic approaches are irreplaceable for advanced NSCLC.^3^ Paclitaxel is a taxane antimitotic agent currently used as the first-line standard therapy regimen with platinum agent for advanced NSCLC.^4, 5, 6^ In addition, paclitaxel as a single agent has also shown efficacy in the second-line setting in advanced and metastatic NSCLC.^5, 6^ However, acquired resistance inexorably develops after a period of treatment.^7^ Paclitaxel resistance can be achieved through several mechanisms, including tubulin isoforms/mutations and the alteration of drug efflux pumps.^7, 8^ Other mechanisms of resistance have also been identified, including deregulation of apoptotic signaling pathways and activation of hypoxia-induced factor 1 (HIF-1) signaling.^9, 10, 11^ Unfortunately, in spite of these advances, treatment of paclitaxel-resistant patients remains a critical clinical challenge. Thus, there is an intense need to further understand mechanisms of paclitaxel resistance.

Epigenetic changes, which are somatically inherited through cell division, are considered as the potential drivers of drug resistance in cancer.^12^ It has been demonstrated that the high rate of epigenetic change in tumors generates diversity in gene expression patterns that can rapidly evolve through drug selection during treatment, leading to the development of acquired resistance.^12^ Histone acetylation, which is the result of the balance between the activity of histone deacetylases (HDACs) and histone acetyltransferases (HATs), is recognized as an important epigenetic event.^13^ The acetylation of histones in nucleosomes contributes to changes in chromatin conformation and mediates to regulation of gene expression.^14^ A lot of studies verified that the aberrantly overexpressed HDACs in various tumors leading to carcinogenesis, cancer progression, and clinical poor outcome.^15, 16, 17^ Thus, HDACs can be a therapeutic intervention for cancer treatment to reverse aberrant epigenetic states associated with cancer, especially drug resistance.^12, 18^ In fact, several studies have illustrated that the synergistic therapeutic effects have been obtained from HDAC inhibitors in combination with DNA-damaging agents, taxanes, targeted agents, death receptor agonists, and hormonal therapies.^18^ Furthermore, HDAC inhibitors have also been found to reverse cytotoxic and targeted agent resistance in various tumors.^18, 19, 20^ However, whether HDACs are implicated in paclitaxel resistance in NSCLCs is still not elucidated. Recently, a clinical study showed that vorinostat, an FDA-approved HDACs inhibitor, enhanced the efficacy of paclitaxel combined with carboplatin in patients with advanced NSCLC,^21^ suggesting that HDACs might have an important role in paclitaxel resistance.

Here, we disclosed that paclitaxel-resistant NSCLC cells displayed the enhanced HDAC activity, increased HDAC1 expression, and raised cell proliferation ability *in vitro* and *in vivo*, and HDAC1 knockdown could sensitize resistant cells to paclitaxel *in vitro*. Clinically, patients owing higher HDAC1 with lower p21 expression tumors showed chemotherapy resistance and bad outcome while taking paclitaxel as a chemotherapy agent. In addition, we also identified a novel HDACs inhibitor, SNOH-3, which was more potent than FDA-approved HDAC inhibitor SAHA to inhibit HDAC activity, induce cell apoptosis, and suppress cell migration, invasion and angiogenesis. Furthermore, when SNOH-3 combined with paclitaxel could overcome paclitaxel resistance through inhibiting HDAC activity, leading to the induction of apoptosis and suppression of angiogenesis. Our results not only identify a therapeutic strategy to abrogate acquired resistance to paclitaxel in NSCLC, but also imply that HDAC1 along with p21 might be a predictive biomarker for paclitaxel treatment.

## Results

### Paclitaxel-resistant NSCLC cells showed enhanced HDAC activity and tumorigenicity

**Figure 1:**
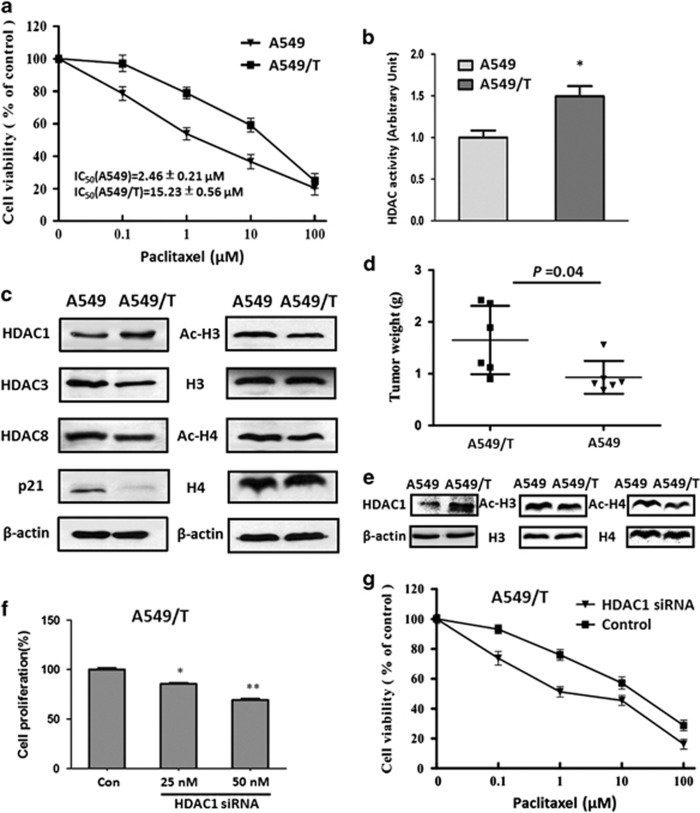
HDAC activity and function in paclitaxel-resistant NSCLC cells. (**a**) The effect of paclitaxel on the cell viability of A549 and A549/T cells. The cells were treated with various concentrations of paclitaxel for 48 h. (**b**) The HDAC activity in A549 and A549/T cells. (**c**) HDAC1, HDAC3, HDAC8, acetylated Histone-3 (Ac-H3), Histone-3(H3), acetylated Hstone-4 (Ac-H4), Histone-4 (H4), and p21 were measured in A549 and A549/T cell lines. *β*-Actin expression was used as a loading control. (**d**) The tumor weight of A549 and A549/T xenograft mouse. (**e**) HDAC1, Ac-H3, H3, Ac-H4, and H4 were measured in A549 and A549/T tumor tissues. *β*-Actin expression was used as a loading control. (**f**) The proliferation rate of A549/T cells after treated with different concentrations of HDAC siRNA (25 and 50 nM) for 48 h. (**g**) The effect of knockdown of HDAC1 by siRNA (50 nM) on the sensitivity to paclitaxel in A549/T cells. *P<0.05, **P<0.01 compare with control

To study the phenomenon of relapse after paclitaxel therapy, we generated *in vitro* chemoresistant model using the NSCLC cell line A549. Drug-resistant cells were established by exposure to increasing concentrations of paclitaxel, and resistance was validated by cell viability. As shown in Figure 1a, as compared with parental cells, A549/T cells exhibited an increased resistance to paclitaxel, with the resistance index is 6.19. In view of the crucial role of HDAC in drug resistance of various tumors,^12^ we also detected the HDAC activity of A549/T and its parental cells. Our results indicated that HDAC activity was increased to 1.5-fold in A549/T cells when compared with that of parental A549 cells (see Figure 1b). Furthermore, we also measured the expression levels of HDACs isoforms, including HDAC1, HDAC3, and HDAC8, in this paired cell lines. As indicated in Figure 1c, only HDAC1 showed an increasing expression in A549/T cells in comparison with parental cells, suggesting that HDAC1 is a dominant factor for HDAC activity in the acquired paclitaxel-resistant process. In consistence with the increasing HDAC activity, its substrates, including p21, acetylated Histone 3 (Ac-H3), and acetylated Histone 4 (Ac-H4), were also changed in A549/T cells (see Figure 1c), suggesting that HDAC activity is increased in A549/T cells.

To further explore the malignant phenotype characteristics of paclitaxel-resistant cells, A549/T and its parental cells were subcutaneously injected into the SCID mice. Our data showed that A549/T cells owned an enhanced tendency to tumorigenicity as compared with A549 cells (*P*=0.04, Figure 1d). Simultaneously, A549/T tumors exhibited an increasing HDAC1 expression and a decreasing Ac-H3 and Ac-H4 level (Figure 1e), which indicated that paclitaxel-resistant cells were critically dependent on HDAC activity. In addition, we found that knockdown of HDAC1 by specific siRNA could inhibit the cell viability and sensitize A549/T cells to paclitaxel (Figures 1f and g). The above data demonstrate that the increasing HDAC activity results in an enhanced tumorigenicity, a greater proliferation ability, and an increased resistance to paclitaxel in NSCLC cells, giving the rationale for an efficacious therapeutic strategy.

### NSCLC tissues with increased HDAC1 and decreased p21 expressions predicted to poor prognosis of patients treated with paclitaxel

**Figure 2:**
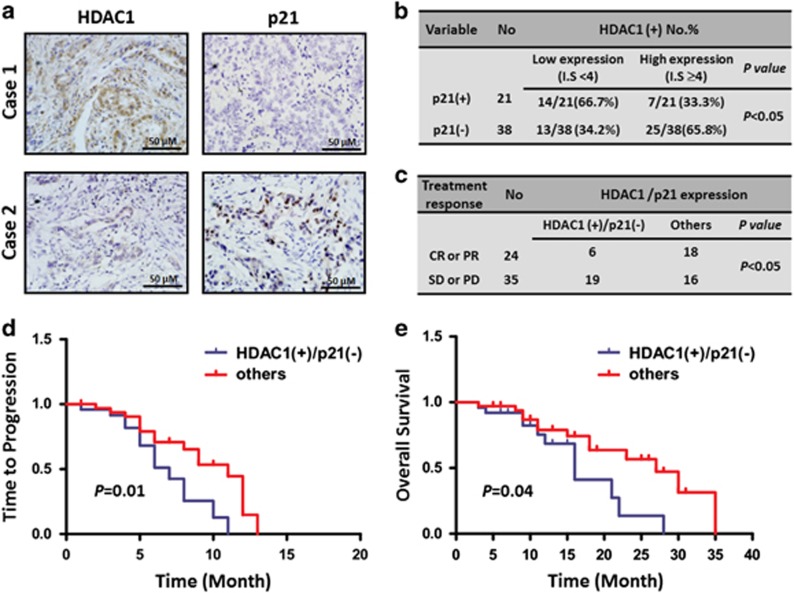
HDAC1 and p21 expression in NSCLCs treated with paclitaxel. (**a**) The expression of HDAC1 and p21 in representative NSCLC tissues. Figures magnified × 400. (**b**) The correlation between HDAC1 and p21 in NSCLC. A sample is defined as HDAC1+ if it has an IS of ≥4; p21+ if it has an IS of ≥2. (**c**) The correlation between treatment response and the expression of HDAC1 along with p21 in NSCLC. CR, complete response; PR, partial response; SD, stable disease; PD, progressive disease. (**d**) TTP according to expression of HDAC1 along with p21 in NSCLC. (**e**) Overall survival according to expression of HDAC1 along with p21 in NSCLC

We next investigated whether the increasing HDAC activity was detectable in human paclitaxel-resistant NSCLC tissue samples. Using immunohistochemistry method, we detected HDAC1 and p21, which was considered as a HDAC target^22^ and was verified to be altered in our *in vitro* data, in paraffin-embedded tissues from 59 paclitaxel-treated NSCLC patients. Clinico-pathological data indicated that there were no relationship between the expressions of HDAC1 or p21 and major clinico-pathological factors (Supplementary Table 1). Additionally, our data showed that there were 32 tumors (54.2%) showing a higher level of HDAC. Interestingly, among 32 tumors with higher HDAC1, 25 tumors (78.1%) expressed p21 at a lower level (Figure 2a). Statistical analysis data indicated that there was a negative correlation between HDAC1 and p21 in lung cancer tissues (*P*<0.05, Figure 2b). Importantly, the opposite expression pattern (higher HDAC1 and lower p21) correlated with bad treatment response, short time to tumor progression (TTP) and poor overall survival than other groups in lung cancer (Figures 2c–e). Therefore, HDAC1 along with p21 may serve as a predictive marker for paclitaxel efficacy and their changes may be associated with paclitaxel resistance.

### SNOH-3, a novel HDAC inhibitor, induced acetylation of histones and prompted upregulation of p21 in NSCLC cells

**Figure 3:**
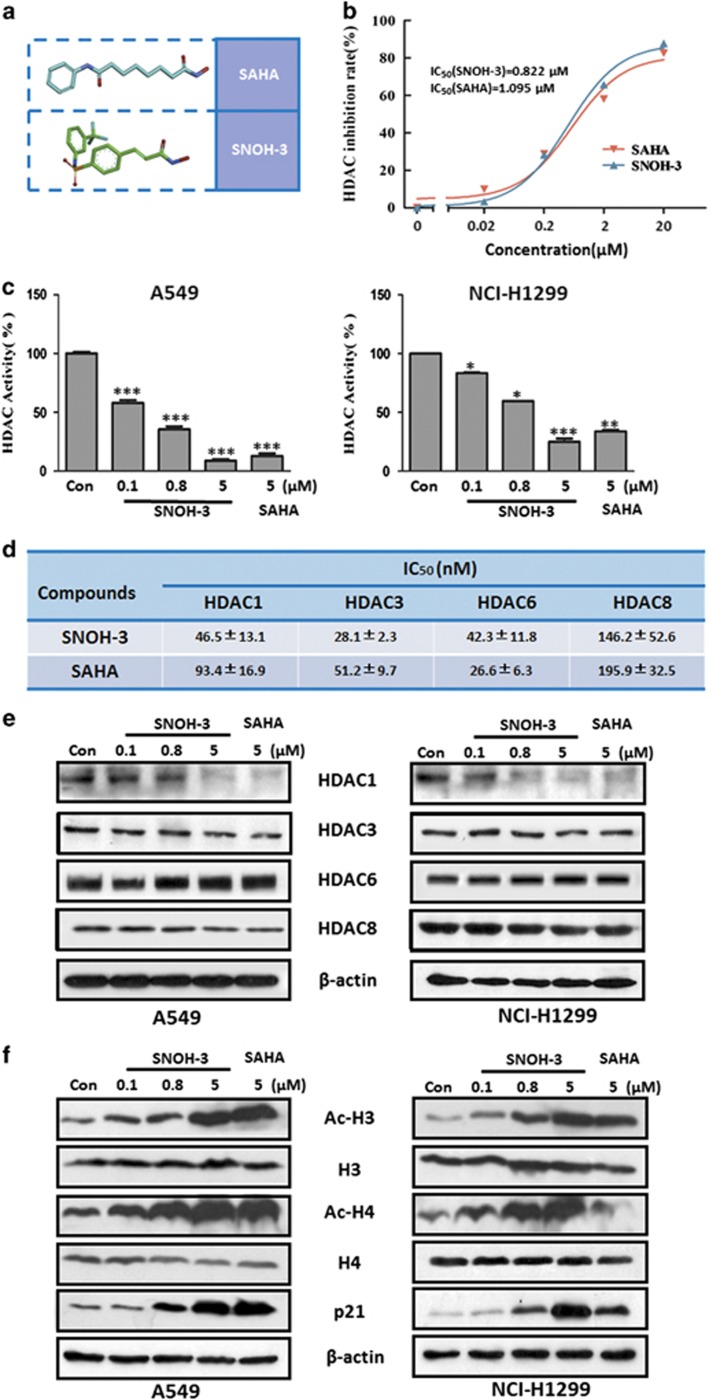
The effects of SNOH-3 on HDAC inhibition. (**a**) 3D structure of SAHA and SNOH-3. (**b**) *In vitro* inhibition of HDAC by SNOH-3 and SAHA at different concentrations (0.02, 0.2, 2, and 20 *μ*M) in cell-free assay. (**c**) The inhibition of HDAC by various concentrations of SNOH-3 (0.1, 0.8, and 5 *μ*M) and SAHA (5 *μ*M) in cell-based assay. (**d**) *In vitro* inhibition of HDAC isoforms by SNOH-3 and SAHA at different concentrations (0.01, 0.1, 1, and 10 *μ*M) in cell-free assay. (**e**) The effects of SNOH-3 on the expression of HDAC1, HDAC3, HDAC6, and HDAC8 in A549 and NCI-H1299 cells after SNOH-3 (0.1, 0.8, and 5 *μ*M) treated for 24 h. (**f**) The effects of SNOH-3 on the expression of Ac-H3, H3, Ac-H4, H4, and p21 in A549 and NCI-H1299 cells after SNOH-3 (0.1, 0.8, and 5 *μ*M) treated for 24 h. *β*-Actin expression was used as a loading control. All error bars are S.E.M. *P<0.05, **P<0.01, ***P<0.001 compare with control

We had synthesized a series of HDAC inhibitors (unpublished data). Among them, SNOH-3 exhibited clear inhibitory effects on HDAC activity *in vitro* with similar capacity to SAHA (Figures 3a and b, preparation of SNOH-3 was provided in Supplementary Materials and Methods). To confirm its inhibition in cells, A549 and a known drug-resistant cells NCI-H1299 treated with SNOH-3 or SAHA were analyzed by cell-based HDAC activity. The data showed that SNOH-3 could suppress activity of HDAC in a concentration-dependent manner in both cell lines and displayed a similar potential to SAHA (Figure 3c).

To further explore the characteristics of SNOH-3 on HDAC inhibition, the inhibitory effects of SNOH-3 on HDAC isoforms activity were determined. As shown in Figure 3d, for HDAC-1, -3, and -8, SNOH-3 exhibited an enhanced inhibitory potential when compared with that of SAHA. However, for HDAC-6, SNOH-3 displayed a lower inhibitory ability in comparison with that of SAHA. Moreover, we also detected the expression level of HDAC isoforms in A549 and NCI-H1299 cells after treated with both compounds. As shown in Figure 3e, SNOH-3 treatment could result in a concentration-dependent decrease of HDAC1 in both cell lines, suggesting that SNOH-3 could inhibit HDAC1 activity through downregulating its expression. In contrast to HDAC1, other isoforms, including HDAC3, -6, and -8, were not obviously changed by treatment with SNOH-3 in both cell lines, indicating that the activity inhibition of HDAC-3, -6, and -8 by SNOH-3 may be not related to their expression regulation.

We subsequently confirmed the HDAC inhibition activity of SNOH-3 by detecting histones acetylation and p21 induction in both cell lines. Parallel to its HDAC inhibition, SNOH-3 significantly increased the levels of acetylated H3, acetylated H4 and p21 (Figure 3f) in a concentration-dependent manner in both cell lines. At the same concentration, SNOH-3 exhibited a greater capability to induce the acetylation than SAHA, especially in NCI-H1299 cells. The above data demonstrate that SNOH-3 is identified as a novel HDAC inhibitor with greater capability than SAHA.

### SNOH-3 induced cancer cell apoptosis and inhibited migration, invasion and tube formation of endothelial cells

**Figure 4:**
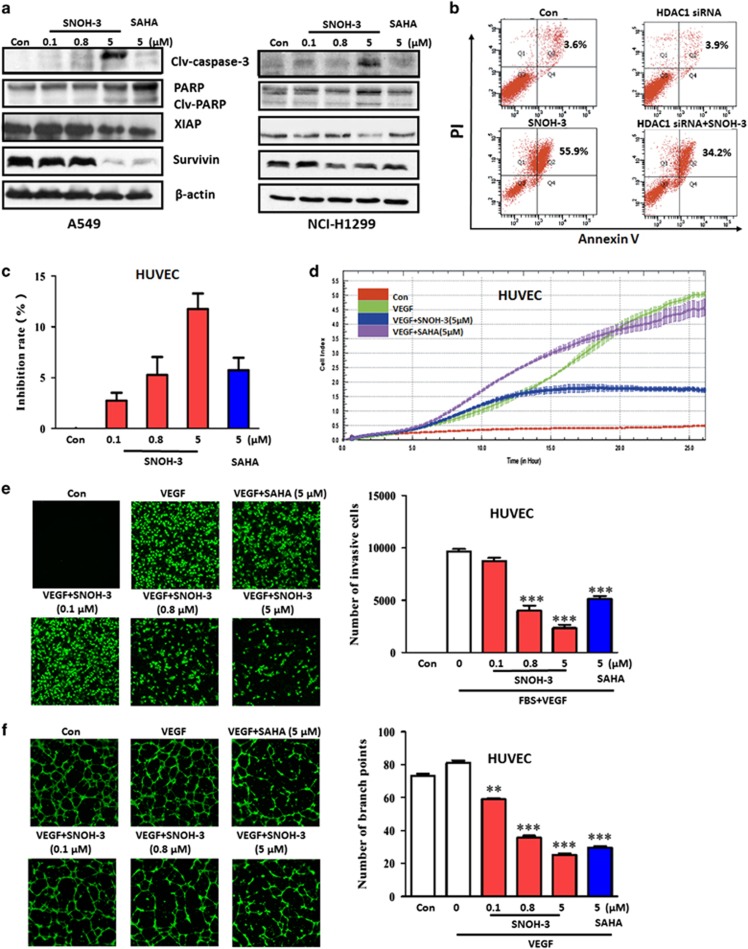
The effects of SNOH-3 on apoptosis of tumor cells and VEGF-induced migration, invasion, and tubular structure formation of endothelial cells. (**a**) The apoptosis-related proteins (clv-caspase-3, PARP, XIAP, and Survivin) were assessed by western blot analyses in A549 and NCI-H1299 cells after SNOH-3 (0.1, 0.8, and 5 *μ*M) treated for 48 h. *β*-Actin expression was used as a loading control. (**b**) The apoptotic cells were assessed by FACS analyses in A549 cells after SNOH-3 (5 *μ*M) or/and HDAC1 siRNA (50 nM) treated for 48 h. (**c**) The effect of SNOH-3 on cell viability of HUVECs. The HUVECs were treated with SNOH-3 (0.1, 0.8, and 5 *μ*M) or SAHA (5 *μ*M) for 24 h. (**d**) HUVECs migration was measured by RTCA assay after SNOH-3 (5 *μ*M) or SAHA (5 *μ*M) treated for 24 h. (**e**) HUVECs invasion was measured by transwell assay after SNOH-3 (0.1, 0.8, and 5 *μ*M) or SAHA (5 *μ*M) treated for 24 h. (**f**) HUVECs tube formation was measured after SNOH-3 (0.1, 0.8, and 5 *μ*M) or SAHA (5 *μ*M) treated for 24 h. All error bars are S.E.M. **P<0.01, ***P<0.001 compare with control or VEGF control

To assess the antitumor effect of the novel HDAC inhibitor SNOH-3, we next examined the effects of SNOH-3 on cell apoptosis by measuring the cleavages of Caspase-3 and PARP, which are regarded as biomarkers of cell apoptosis.^23^ Our results indicated that SNOH-3 treatment at higher concentration (5 *μ*M) resulted in an obvious increase in the cleavages of Caspase-3 and PARP as compared with control, but there were no significant effects of SNOH-3 at lower concentrations (0.1 and 0.8 *μ*M, Figure 4a). In addition, SNOH-3 exposure also resulted in diminished expression of anti-apoptotic proteins XIAP and Survivin, which might explain the underlying mechanisms of SNOH-3-induced apoptosis. The above data were further confirmed using Annexin V/PI double staining methods. The data revealed that SNOH-3 (5 *μ*M) caused an increase in the percentage of apoptosis cells (Annexin V+) in A549 cells (Figure 4b). The role of HDAC inhibition in SNOH-3-induced apoptosis was confirmed by silencing HDAC1 in A549 cells. The SNOH-3-caused increase in apoptosis was partially blocked by specific HDAC1 siRNA (Figure 4b).

The migration, invasion, and angiogenesis of endothelial cell are inevitable to solid tumor growth and metastasis.^24^ Therefore, we also evaluated the effects of SNOH-3 on VEGF-induced migration, invasion, and tube formation of human umbilical vein cells (HUVECs) *in vitro*. To exclude the cytotoxicity of SNOH-3, we first examined the effect of SNOH-3 on the viability of HUVECs. SNOH-3 treatment for 24 h caused a slight decrease in the percentage of viable HUVECs at 5 *μ*M with inhibition rate lower than 15% (Figure 4c). Thus, the maximal concentration of 5 *μ*M was determined in the following experiments. The effect of SNOH-3 on the migration of HUVECs was detected by real-time cell analysis (RTCA) assay. Our data indicated that SNOH-3 obviously reduced VEGF-induced HUVECs migration (Figure 4d), whereas SAHA did not display an inhibitory effect. In addition, transwell invasion assay showed that SNOH-3 dramatically suppressed VEGF-induced HUVECs invasion (Figure 4e). Next, we investigated how SNOH-3 affected HUVEC tube formation. As shown in Figure 4f, in the presence of VEGF, elongated and robust tube-like structures were formed. When exposed to SNOH-3, the formation of tubular structures, especially branch points, was significantly inhibited. Both inhibitory effects of SNOH-3 were concentration dependent, and SNOH-3 exhibited a relatively enhanced ability to inhibit invasion and tube formation of HUVECs as compared with SAHA.

To explore the underlying mechanism of SNOH-3-caused inhibition of migration, invasion, and tube formation in HUVECs, we measured the expression level of VEGF in HUVECs after exposure to SNOH-3. The results revealed that the protein expression of VEGF was downregulated by SNOH-3 treatment in HUVECs (Supplementary Figure 1).

Previous study reported that KLF4, a transcription repressor of VEGF,^25^ was regulated by HDAC activity,^26^ thus we also assessed the effect of SNOH-3 on the expression of KLF4 in HUVECs. Our data indicated that SNOH-3 could increase the expression of KLF4 in a concentration-dependent manner in HUVECs (Supplementary Figure 1). These results indicated that SNOH-3 might suppress cell migration, invasion, and angiogenesis through the induction of KLF4 expression to reduce VEGF.

### SNOH-3 combined with paclitaxel overcome paclitaxel resistance *in vitro* via inducing apoptosis

**Figure 5:**
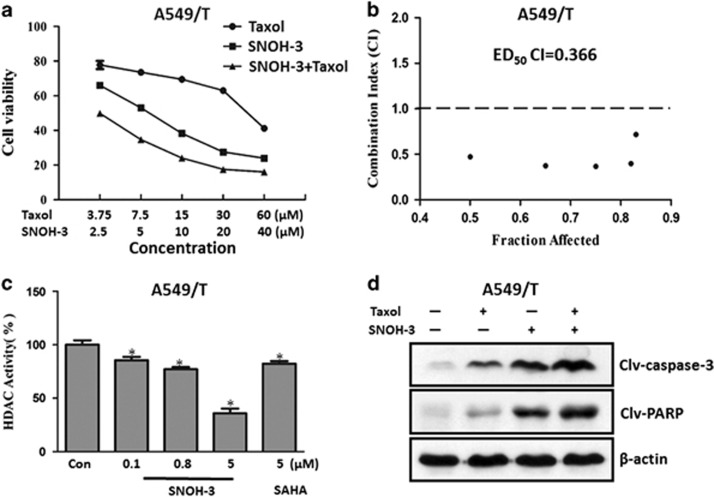
The effects of SNOH-3, paclitaxel, and their combination on the growth and apoptosis of A549/T cells. (**a**) The growth curve of A549/T cells after treated with SNOH-3, paclitaxel, and the combination of SNOH-3 and paclitaxel. (**b**) Analysis of the combination of SNOH-3 and paclitaxel in A549/T cells. The cells were treated for 48 h using increasing concentrations of SNOH-3 and paclitaxel, either alone or in a fixed ratio. The resultant data were analyzed using Calcusyn program, and graphs from the averaged results of three independent experiments are shown. (**c**) The HDAC activity of A549/T cells was measured by cell-based HDAC assay after SNOH-3 (0.1, 0.8, and 5 *μ*M) or SAHA (5 *μ*M) treated for 24 h. (**d**) The apoptosis was assessed by western blotting after SNOH-3 (10 *μ*M) or/and SAHA (15 *μ*M) treated for 48 h. Cleavages of caspase-3 and PARP were considered as apoptosis. *P<0.05 compare with control

Because HDAC1 was overexpressed in paclitaxel-resistant A549 (A549/T) cells and its silence could sensitize A549/T cells to paclitaxel, the growth inhibitory effect of SNOH-3 in combination with paclitaxel on A549/T cells was assessed. As shown in Figure 5a, the combination of SNOH-3 and paclitaxel could obviously inhibit cell proliferation as compared with SNOH-3 or paclitaxel alone. The synergistic action analysis data indicated that the combination led to a strong synergistic antiproliferative activity on A549/T cells (Figure 5b), and the ED_50_ CI was 0.366. In addition, we also found that SNOH-3 treatment could suppress the HDAC activity in a concentration-dependent manner in A549/T cells (Figure 5c), suggesting that HDAC inhibition might have a crucial role in this synergistic effect.

Corresponding to the synergistic inhibition on cell proliferation, SNOH-3 plus paclitaxel could result in an increased cleavages of PARP and caspase-3 as compared with paclitaxel or SNOH-3 alone (Figure 5d), suggested that the synergistic antiproliferation effect of SNOH-3 and paclitaxel on A549/T cells might be due to induction of apoptosis.

### SNOH-3 combined with paclitaxel abrogated paclitaxel resistance *in vivo* through inducing apoptosis and suppressing angiogenesis

**Figure 6:**
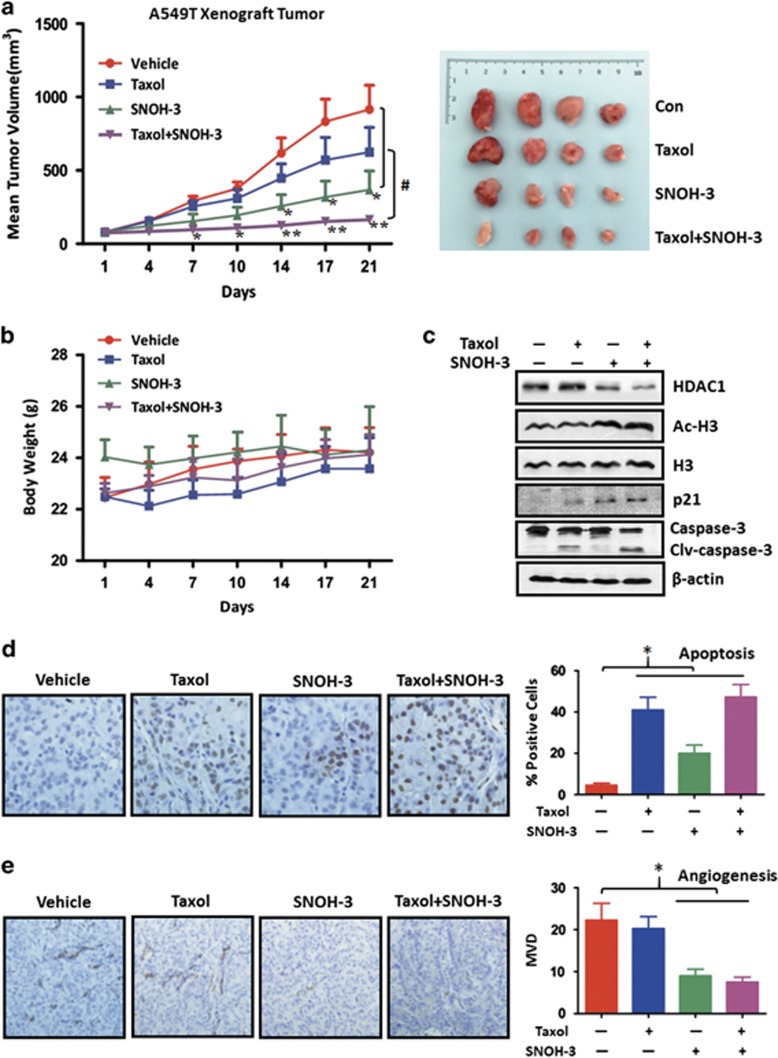
The antitumor effect of SNOH-3 and paclitaxel on A549/T human xenograft models. (**a**) The mice transplanted with A549/T human xenografts were randomly divided into four groups and given injection of SNOH-3 (40 mg/kg/3 days), paclitaxel (10 mg/kg/week), and the combination or vehicle for a period of 3 weeks. The tumor volumes are expressed as mean±S.D. (*n*=4 per group). (**b**) The average body weight of each group is expressed as mean±S.D. (*n*=4 per group). (**c**) Expression of HDAC1, Ac-H3, H3, p21, caspase-3, and PARP extracted from the tumor tissues of drug-administrated mice of four groups was detected by western blotting. (**d**) The effects of SNOH-3, paclitaxel, and the combination on apoptosis of A549/T xenograft tumors were measured by TUNEL. (**e**) The effects of SNOH-3, paclitaxel, and the combination on MVD of A549/T xenograft tumors were measured by immunohistochemistry. All error bars are S.E.M. *P<0.05, **P<0.01, SNOH-3, paclitaxel single administration or the combination administration compared with the control, ^#^*P*<0.05, the combination administration compared with SNOH-3, paclitaxel single administration or vehicle

We next evaluated the effects of this combination strategy *in vivo*. NOD/SCID mice bearing A549/T xenografts were treated with paclitaxel alone, SNOH-3 alone, or dual combinations (SNOH-3 plus paclitaxel). Xenografts treated with paclitaxel showed a weak inhibition, which verifying a resistant characteristic (Figure 6a). On the contrary, SNOH-3 administration could result in an obvious suppression of tumor growth during the course of the experiment (2 weeks, Figure 6a). Remarkably, mice treated with the dual combination exhibited a robust inhibition of tumor growth, compared with mice treated with SNOH-3 and paclitaxel alone, therefore mirroring our *in vitro* results (Figure 6a). In addition, all mice tolerated the treatment well without significant toxicity and showed stable body weights (Figure 6b). Inhibitory effect of SNOH-3 and dual combination on HDAC was confirmed by testing the expression levels of HDAC1, Ac-H3, and p21 in tumor cells obtained from xenografts (Figure 6c). Moreover, in agreement with our *in vitro* results, xenografts treated with the dual combination displayed an increased apoptosis (cleaved caspase-3 and terminal deoxynucleotide transferase-mediated dUTP nick end labeling (TUNEL), Figures 6c and d) and a decreased angiogenesis (MVD, Figure 6e) in comparison with paclitaxel or SNOH-3-treated animals (Figures 6c and d). These results indicated that SNOH-3 combined with paclitaxel could overcome paclitaxel resistance *in vivo* via the inhibition of HDAC activity to induce apoptosis and suppress angiogenesis.

## Discussion

Based on demonstrated favorable risk-benefit profiles, paclitaxel remains a key component in the first-line standard of care for advanced NSCLC.^5^ However, the emergence of acquired resistance is inevitable and remains the major obstacle. Delineation of these resistance mechanisms will pave the way for rationally designed and hopefully long-lasting combinations of targeted therapies. In the present study, we found that HDAC activation inhibits its targets expression, such as p21, in paclitaxel-resistant NSCLC cells that lead to the enhancement of proliferation and tumorigenesis, thus dampening the response to the paclitaxel. Furthermore, in clinical NSCLC specimens, HDAC activity was increased in paclitaxel-treated tumors with drug resistance and poor survival. Importantly, our results indicate that HDAC1 inhibition could be a strategy to overcome paclitaxel-acquired resistance. Overall, our results have important implications for clinical oncology and lung cancer biology.

Until now, there were 18 mammalian HDAC isoforms have been identified in humans, which could be divided into four classes according to their homology to yeast prototypes.^14^ Among all of the HDACs, HDAC1, -3, -6, and -8 have been frequently revealed to be closely related to tumorigenesis.^14^ Here, we found that HDAC activity was increased in paclitaxel-resistant NSCLC cells in comparison with parental cells, and that resulted in an enhanced proliferation and tumorigenesis capability, suggesting a novel biology role of HDAC in drug resistance. Furthermore, the prognosis significance of HDAC in paclitaxel administrated NSCLCs implies that HDAC might serve as a predictive biomarker for paclitaxel treatment. Interestingly, our data showed that only HDAC1, but not HDAC3 and HDAC8, was overexpressed in A549/T cells, which displayed an increased HDAC activity. This could be explained by the following fact: first, several studies have demonstrated that HDAC1 is a dominant isoform in determining HDAC activity.^27, 28^ Second, there are several literatures showed that HDAC1 was overexpressed in multidrug resistance cells.^19, 29, 30^ Third, loss of HDAC1 usually results in a compensatory increase in the related HDACs.^27^ So it is possible that HDAC1 overexpression would result in the loss of other HDAC isoforms. However, it will be important to fully understand what dictates drug resistance by this understudied HDAC1. Much remains to be determined regarding the inter-regulation of HDAC isoforms in the process of paclitaxel resistance.

The crucial role of HDAC in NSCLC paclitaxel resistance in our *in vitro*, *in vivo*, and clinical tissues studies and limitation of FDA-approved HDAC inhibitors in solid cancer prompted us to develop novel HDAC inhibitors. Here, we developed a novel HDAC inhibitor, SNOH-3, which showed more antitumor potent than SAHA. For total activity of HDACs, SNOH-3 showed a similar potent to SAHA, whereas for HDAC1 and HDAC3, SNOH-3 exhibited a greater capability than SAHA. The enhanced capability also was presented in the activation of its substrates. In comparison with SAHA, SNOH-3 displayed a greater ability to increase the expression of Ac-H3, Ac-H4, and p21 in NSCLC cells, especially in NCI-H1299 cells, which is considered as a drug-resistant cell line. In consistent with HDAC activity data, the functional studies also indicated that SNOH-3 induced a greater apoptosis in NSCLC cells when compared with those of SAHA, and HDAC1 had an important role in this process. Mechanistically, we found that SNOH-3 reduces the anti-apoptosis protein Survivin and XIAP, which could be explained by the fact that Survivin and XIAP are also the targets of HDAC inhibitor,^31, 32^ so it is possible that SNOH-3 induces apoptosis through reducing the expression of Survivin and XIAP. Furthermore, our results showed that SNOH-3 exhibited an obvious inhibition to VEGF-induced migration, invasion, and angiogenesis in HUVECs. The underlying mechanisms might be that SNOH-3 treatment resulted in HDAC inhibition, then led to upregulation of KLF4 which is considered as a transcription repressor of VEGF,^25, 26^ finally contributed to the downregulation of VEGF.

Among the mechanisms of paclitaxel resistance, alteration of drug target (tubulin), overexpression of drug efflux pumps, and activation of HIF-1 signaling are widely recognized. Here, we found that HDAC was also involved in the paclitaxel resistance. The inner link between HDAC and the classical resistant mechanisms might be illustrated by the previous literatures: (1) Dowdy *et al.*^33^ reported that TSA, an HDAC inhibitor, stabilized microtubules via *α*-tubulin acetylation in endometrial cancer cells and enhanced paclitaxel effects. (2) Kaewpiboon *et al.*^34^ demonstrated that P-glycoprotein (Pgp), a well-known drug efflux pump, could be regulated by HDAC at the transcriptional level in lung cancer cells. (3) Several groups showed that HDAC inhibitors could inhibit HIF-1 activity by modulating its interactions and its acetylation status.^35, 36^ The above-reported results indicated that HDAC might be also involved in other paclitaxel-resistant mechanisms. However, the detail role of HDAC in these process need be further illustrated.

Importantly, although resistance to paclitaxel in NSCLC has been a subject of considerable interest, the development of therapeutic strategies that overcome paclitaxel resistance has remained an elusive challenge in clinical oncology. The increase of HDAC activity in our *in vitro* and *in vivo* models prompted us to evaluate the combination strategy that abrogated paclitaxel resistance *in vitro* and *in vivo*. Our data indicated that SNOH-3 in combination with paclitaxel exerted promising activity to overcome paclitaxel resistance *in vitro* and in preclinical models through induction apoptosis and inhibition angiogenesis. These data corroborate previous study in clinical, which have found an enhanced efficacy of HDAC inhibitor combined with carboplatin and paclitaxel in patients with advanced NSCLC.^21^ In summary, given the limited capacity of paclitaxel to control advanced NSCLC, this work lays the foundation for a promising therapeutic strategy.

In conclusion, a common and devastating phenomenon in clinical oncology is acquired drug resistance. We provide evidence that the increase in HDAC activity was found from NSCLC with paclitaxel-acquired resistant cells, and HDAC1 overexpression along with p21 downregulation predicted bad response, short TTP, and poor overall survival of NSCLC patients with paclitaxel treatment. Knockdown of HDAC1 could sensitize paclitaxel-resistant cells to paclitaxel and inhibit cell proliferation. The combination of HDAC inhibitor SNOH-3 with paclitaxel inactivated HDAC, and thus conferred its proapoptotic and antiangiogenic effects in paclitaxel-resistant models. Our finding not only elucidated an additional mechanism for acquired paclitaxel resistance in NSCLC, but also provided a promising therapeutic strategy to overcome paclitaxel-acquired resistance.

## Materials and Methods

### Cell lines and cell culture

Human lung cancer cell lines A549 and NCI-H1299, and HUVEC were obtained from the American Type Culture Collection (Manassas, VA, USA). A549/paclitaxel resistance cells (A549/T) were obtained from Key GEN Bio Tech (Nanjing, China, CHN). These cancer cells were routinely cultured in RPMI-1640 or MEM supplemented with 10% fetal bovine serum (FBS) and maintained at 37 °C in a humidified incubator with 5% CO_2_. Specially, the resistance of A549/T cells was maintained by continuously exposing to paclitaxel (200 ng/ml). HUVECs were maintained as a monolayer in MCDB131 medium supplemented with 20% (v/v) FBS, 1% (v/v) l-glutamine, 5 units/ml heparin, and 50 mg/ml endothelial cell growth factor (ECGF) using culture flasks or plates precoated with 1% (v/v) gelatin.

### Patients and chemotherapy

A total of 56 patients with advanced NSCLC (stage IIIB and stage IV) were enrolled between January 2004 and June 2012 from Wuhan General Hospital of Guangzhou Command (Wuhan, PR China). The enrolled patients met the following eligibility criteria: histological or cytological confirmation of NSCLC, the presence of measurable disease, no adjuvant or neoadjuvant therapy and surgery, no second malignancies, availability of adequate diagnostic tumor tissue (taken from brochoscopic biopsy, percutaneous lung biopsy or metastatic sites). The clinicopathologic features of these patients have been summarized in Supplementary Table 1.

Each patient underwent the treatment with at least two cycles of the first-line platinum–paclitaxel chemotherapy. Response to treatment was determined after 2–3 cycles by RECIST (Response Evaluation Criteria in Solid Tumors) criteria, which classified the responses into complete response (CR), partial response (PR), stable disease (SD), and progressive disease (PD). SD plus PD were considered as chemotherapy resistance. TTP was defined as the time between the onset of chemotherapy and the date of first documented disease progression. Overall survival (OS) was defined as the time between the onset of chemotherapy and the date of the last follow-up or death from any cause. Ethical oversight and approval was obtained from the Institutional Review Board of Wuhan General Hospital of Guangzhou Command.

### Immunohistochemistry

A tissue microarray (TMA) was constructed (in collaboration with the Shanghai Biochip Company Ltd., Shanghai, China, CHN) as described previously.^17^ Following antigen retrieval and blocking, TMA sections (4 mm) were immunostained using antibodies against HDAC1 (1 : 200 dilution; Abcam, Cambridge, UK) and p21^Waf1/Cip1^ (1 : 50 dilution; Cell Signaling Technology, Danvers, MA, USA) and with detection using the avidin–biotin complex method (DAKO) visualized by 3,3′-diaminobenzidine (DAB). Slides were lightly counterstained with hematoxylin. The evaluation of both the intensity of immunohistochemical staining and the proportion of positively stained epithelial cells was previously described.^33^

### Compounds and reagents

SNOH-3, with purity >98%, was synthesized in Medicine Chemistry Laboratory at Shenyang Pharmaceutical University (see Figure 3a and Supplementary data). SAHA were obtained from Sigma (St. Louis, MO, USA). These agents were dissolved in DMSO to 100 mM and stored at −20 °C. Before treatment, the stock solution is diluted to different concentrations. The final concentration of DMSO in cultures is 0.1% (v/v) or less. MTT (3-(4,5-dimethylthiazol-2-yl)-2,5-diphenyl tetrazolium bromide) and propidium idodide (PI) were purchased from Sigma. The primary antibodies against HDAC1, HDAC3, HDAC6, HDAC8, Histone 3, Histone 4, p21^WAF1/CIP1^, PARP, Caspase-3, Survivin, XIAP, KLF4, VEGF, and *β*-actin were got from Cell Signaling Technology. The primary antibodies against CD31 were obtained from Santa Cruz Biotechnology (Santa Cruz, CA, USA). The primary antibodies against Ac-H3 and Ac-H4 were purchased from Millipore (Boston, MA, USA). The HDAC1 Silencer Select Validated siRNA was got from Life Technologies (Waltham, MA, USA).

### Cell viability assay

The *in vitro* cell viability was determined by MTT assay. The cells (1 × 10^5^ cells/ml) were seeded into 96-well culture plates. After overnight incubation, the cells were treated with various concentrations of agents for 24 or 48 h. Then 10 *μ*l MTT solution (2.5 mg/ml in PBS) was added to each well, and the plates were incubated for an additional 4 h at 37 °C. After centrifugation (2500 r.p.m., 10 min), the medium with MTT was aspirated, followed by the addition of 100 *μ*l DMSO. The optical density of each well was measured at 570 nm with a Biotek Synergy HT Reader (Winooski, VT, USA).

### HDAC activity assay

The *in vitro* HDAC assay was performed with an HDAC fluorescent activity assay kit (BioVision, San Francisco, CA, USA) as our previous reported.^17^ Briefly, A549 nuclear proteins were incubated with different concentrations of compounds and SAHA (Sigma) at 37 °C for 30 min in the presence of an HDAC fluorimetric substrate. The HDAC assay developer (which produces a fluorophore in reaction mixture) was added, and the fluorescence was measured using a microplate reader (Molecular Devices, Sunnyvale, CA, USA). For cell-based HDAC activity assay, the A549, A549/T, and NCI-H1299 cells were treated with different concentrations of SNOH-3 or SAHA for 24 h before assays. Proteins were isolated by using cell lysis buffer (Beyontime, Guangdong, China, CHN). The protein concentration was measured by BCA protein assay (Beyontime, CHN). The other procedure is same as *in vitro* HDAC assay. HDAC activity is presented as the means±S.E.M. of three determinants.

### HDAC isoforms activity assay

The HDAC1, 3, 6, and 8 activity was assessed using commercial kits (BPS Bioscience, San Diego, CA, USA). Briefly, the purified HDAC proteins were incubated with different concentrations of SNOH-3 and SAHA at 37 °C for 30 min in the presence of an HDAC fluorimetric substrate containing an acetylated lysine side chain. The HDAC assay developer (which produces a fluorophore in reaction mixture) was added, and the fluorescence was measured using a microplate reader (Molecular Devices). HDAC isoforms activity is presented as the means±S.E.M. of three determinants.

### Western blot analysis

About 1 × 10^7^ cells were gathered after pre-treatment for the indicated time periods as described previously. Western blotting was performed as previously described.^17^ Briefly, an equal amount of total protein extracts from cultured cells or tissues was fractionated by 10–15% SDS-PAGE and then electrically transferred onto polyvinylidene difluoride (PVDF) membranes. Mouse or rabbit primary antibodies and appropriate horseradish peroxidase (HRP)-conjugated secondary antibodies were used to detect the designated proteins. The bound secondary antibodies on the PVDF membrane were reacted with ECL detection reagents (Pierce, Rockford, IL, USA) and exposed to X-ray films. Results were normalized to the internal control *β*-actin.

### Flow-cytometry analysis

Analyses for apoptosis were also conducted with an Annexin V–FITC Apoptosis Detection Kit (BioVision). A549 cells (1 × 10^6^) were exposed to SNOH-3 (5 *μ*M) or/and HDAC1 siRNA (50 nM) for 48 h. They were collected by centrifugation and resuspended in 500 *μ*l of 1 × binding buffer. Annexin V–fluorescein isothiocyanate (FITC; 5 *μ*l) and PI (5 *μ*l) were added to the cells. After incubation at room temperature for 5 min in the dark, cells were analyzed by FACS using a flow cytometer (Becton Dickinson, Bergen, NJ, USA). Cells that stained Annexin V–FITC were analyzed.

### Real-time cell analysis

The CIM-plate contains 16 modified Boyden chambers, which can be used independently to measure cell migration in real time through 8 *μ*m pores of a polyethylene terephthalate membrane onto gold electrodes on the underside of the membrane using the xCELLigence analyser system (ACEA Biosciences, San Diego, CA, USA). Experiments were set up according to the manufacturer's instructions with the membrane uncoated (migration). A chemotactic signal for movement was provided by inoculating 30 000–50 000 HUVECs in serum-free medium in the upper chamber and supplying 10% FBS in the lower chamber (10 ng/ml VEGF with the relevant concentration of drug). Cell index (electrical impedance) was monitored every 5 min for the duration of the experiment. The cell index represents the capacity for cell migration, and the slope of the curve can be related to the migration velocity of tumor cells. The cell index thus reflects the tumor cell's migratory capacity.

### Transwell invasion assay

Invasion of HUVECs was assayed using Transwell (Corning Costar, Corning, NY, USA) with 6.5-mm diameter polycarbonate filters (8-*μ*m pore size). Briefly, the lower surface of the filter was coated with Matrigel. Fresh M200 medium (1% FBS) containing 10 ng/ml VEGF was placed in the lower wells. The cells were trypsinized and suspended at a final concentration of 1 × 10^6^ cells/ml in M200 containing 1% FBS. Various concentrations of SNOH-3 and SAHA were given to the cells for 30 min at room temperature before seeding. One hundred microliters of the cell suspension were loaded into each of the upper wells, and the chamber was incubated at 37 °C for 12 h. The cells were fixed and stained with Calcein-AM. Non-invasion cells on the upper surface of the filter were removed by wiping with a cotton swab, and chemotaxis was quantified with a high content drug screening system ImageXpress^R^ Micro (Molecular Devices) by counting cells that had migrated to the lower side of the filter.

### Tube formation assay

HUVECs was incubated in M200 containing 2% LSGS (Low Serum Growth Supplement). Various concentrations of SNOH-3 and SAHA were added to the cells for 30 min at room temperature before seeding. Cells were plated onto the layer of Matrigel at a density of 1.8 × 10^5^ cells per well, followed by the addition of 10 ng/ml VEGF. After 12 h, the cells were fixed and stained with Calcein-AM. The tube branch points were determined using the high content drug screening system ImageXpress^R^ Micro (Molecular Devices) after photographed (× 400).

### Determination of combination index

A549/T cells were treated with different concentrations of single SNOH-3, paclitaxel, or their combination. The cell viability was measured by MTT assay. The nature of the drug interaction was analyzed by using the combination index (CI) according to the method of Chou and Talalay. A CI value lower than 0.90 indicates synergism; a CI value between 0.90 and 1.10 indicates additive; and a CI value higher than 1.10 indicates antagonism. Data analysis was performed by the Calcusyn software (Biosoft, Oxford, UK).

### Mouse xenograft tumors study

For tumorigenesis assessment, the viable A549 or A549/T cells (5 × 10^6^/100 *μ*l PBS per mouse), as confirmed by trypan blue staining, were subcutaneously injected into the right flank of 7- to 8-week-old male SCID mice. After 28 days, the mice were killed and the tumors were excised and stored at −80 °C until western blotting. To determine the *in vivo* antitumor activity of SNOH-3 combined with paclitaxel (Taxol), viable A549/T cells (5 × 10^6^/100 *μ*l PBS per mouse) were subcutaneously injected into the right flank of 7- to 8-week old male SCID mice. When the average tumor volume reached 50 mm^3^, the mice were randomly divided into four treatment groups, including control (saline only, *n*=4), SNOH-3 (40 mg/kg/3 days, i.p.; *n*=4), Taxol (10 mg/kg/week, i.v.; *n*=4), and the combination (*n*=4). Tumor size was measured once every 3 days with a caliper (calculated volume=shortest diameter^2^ × longest diameter/2). Body weight was recorded once every 3 days. After 21 days, the mice were killed and the tumors were excised and stored at −80 °C until further analysis. These studies were performed in strict accordance with the recommendations in the Guide for the Care and Use of Laboratory Animals of the National Institutes of Health. The protocol was approved by the Committee on the Ethics of Animal Experiments of the Shenyang Pharmaceutical University.

### Immunohistochemistry and TUNEL assay

Tissues embedded in paraffin were cut to a section of 4 *μ*m, deparaffinized, and treated with citrate buffer. Then, they were blocked with avidin/biotin for 20 min. The slides were incubated with anti-CD31 for overnight at 4 °C. Next, the slides were treated with secondary antibody with horseradish peroxidase goat anti-rabbit for 1–3 h and developed with 3,3-diaminobenzidine (Sigma-Aldrich, St. Louis, MO, USA). Finally, the slides were counterstained with hematoxylin. The microvessel density (MVD) was determined in a blinded manner by counting the total number of vessels in three fields. TUNEL system (Roche, Basel, Switzerland) was used to detect apoptosis in the tumor sections placed on slides according to the manufacturer's protocol. TUNEL reaction solution was substituted with TdT-free solution for a negative control. Sections were pretreated 10 min with DNase and visualized by DAB staining. Positive nuclei were identified by brown color. The percentage of positive cells over the total cells counted was calculated.

### Statistical analysis

Differences between experimental groups were evaluated by one-way ANOVA or Turkey's *post-hoc* test using the SPSS11.5 software package for Windows (SPSS, Chicago, IL, USA). Survival curves were constructed using the Kaplan–Meier method. Statistical significance was based on a *P*-value of 0.05 (*P*<0.05, two-tailed test).

## Abbreviations

NSCLC: non-small cell lung cancer
HDAC: histone deacetylase
Ac-H3: acetylated Histone 3
Ac-H4: acetylated Histone 4
TTP: time to tumor progression
OS: overall survival
A549/T: paclitaxel-resistant A549
HUVECs: human umbilical vein endothelial cells
CI: combination index
MVD: microvessel density
