# Supplementary figures and images for: Targeting HDAC with a novel inhibitor effectively reverses paclitaxel resistance in non-small cell lung cancer via multiple mechanisms
Source: Cell Death Dis. 2016 Jan 21;7(1):e2063–. doi: 10.1038/cddis.2015.328 (PMC4816165; doi:10.1038/cddis.2015.328)

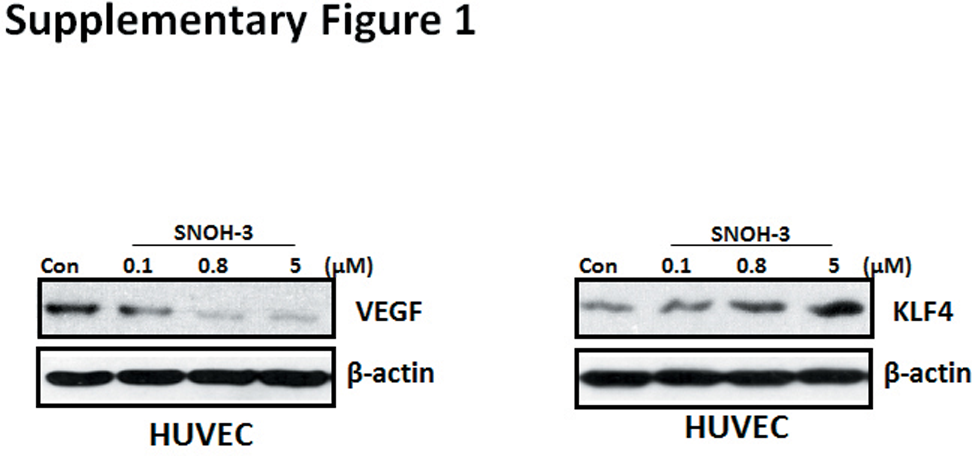

Supplement: Supplementary Figure 1 [file cddis2015328x1.tif]
