# Supplementary material for: Targeting HDAC with a novel inhibitor effectively reverses paclitaxel resistance in non-small cell lung cancer via multiple mechanisms
Source: Cell Death Dis. 2016 Jan 21;7(1):e2063–. doi: 10.1038/cddis.2015.328 (PMC4816165; doi:10.1038/cddis.2015.328)
Supplement: Supplementary Table 1 [file cddis2015328x2.doc]

**Supplementary Materials and Methods**

**Chemistry**

Unless stated otherwise, all reagents and solvents were used as received from commercial suppliers. ^1^H NMR spectra were recorded on a Bruker Ascend 300 MHz spectrometer. The splitting of proton resonances in the ^1^H NMR spectra are defined as s = singlet, d = doublet, t = triplet, and m = multiplet. Electrospray ionization mass spectrometry was carried out with an Agilent 1100 SL instrument using MeOH as solvent.

Fig 1. The synthetic route of SNOH-3

**(2*E*)​-3-​[4-​(chlorosulfonyl)​phenyl]-2-​propenoic acid methyl ester**(**2**)

To neat chlorosulfonic acid (50.1 g, 0.43 mol) at 0℃, (*E*)-cinnamic acid methyl ester (**1**, 8.8 g, 0.054 mmol) was slowly added in portions. The mixture was stirred at 0℃4 h. The dark, viscous syrup was poured onto ice and the mixture was partitioned by AcOEt(3×100 ml) and the combined organic phase was washed successively with H_2_O and saturated NaCl solution, and dried by anhydrous MgSO4. Then the organic phase was evaporated in vaccum and gave 10.3 g the residue(**2**), the yield 73.5%.

**(2*E*)-3-{4-[(Phenylamino)sulfonyl]phenyl}-2-propenoic acid methyl ester** (**3**)

To a mixture of 3-trifluoromethylbenzenamine (4.83 g, 30 mmol) and pyridine (10 ml) in AcOEt(100 ml), a soln. of **2** (7.82 g, 30 mmol) in AcOEt (30 ml) was slowly added, and the resultant soln. was stirred at room temperature for 4 h. The mixture was poured onto cold water and the organic layer was separated, the residue water phase was partitioned by AcOEt (3×100 ml). The combined organic layer was washed successively with H_2_O, saturated NaCl solution, dried by anhydrous MgSO4. Then the organic phase was evaporated in vaccum and gave 10.3 g the residue(**3**), the yield 89.0%.

**(2*E*)-*N*-Hydroxy-3-{4-[(3-trifluoromethylphenylamino)sulfonyl]phenyl}-2-propenamide**(**SNOH-3**)

The mixture of **3**(3.85 g, 10 mmol), hydroxylamine hydrochloride(7.0 g, 100 mmol) and potassium hydroxide(6.0 g, 110 mmol) in methanol(100 ml) was stirred 2 h at room temperature at first, then refluxed for another 6 h. The reaction was completed monitored by TLC. The mixture was evaporated and the residue was added into water. The precipitate filtered, washed with H_2_O, and dried: 2.89 g (75%) of the crude **SNOH-3**. It was recrystallized from EtOH: 2.0 g **SNOH-3** (70%). m.p. 118-120℃。^1^H NMR (DMSO-*d*_6_, 300 MHz)：δ10.88 (1 H, s, CONHOH), 10.81 (1 H, s, CONHOH), 9.16 (1 H, s, SO_2_NH), 7.77 (4 H, q, Ar-H), 7.45 (1 H, d, *J* 15.9 Hz, CH_2_=CH_2_), 7.44 (4 H, m, Ar-H), 6.55 (1 H, d, *J* 15.9 Hz, CH_2_=CH_2_); MS (ESI) m/z 385 [M-H]^-^.
