# Supplementary material for: Targeting HDAC with a novel inhibitor effectively reverses paclitaxel resistance in non-small cell lung cancer via multiple mechanisms
Source: Cell Death Dis. 2016 Jan 21;7(1):e2063–. doi: 10.1038/cddis.2015.328 (PMC4816165; doi:10.1038/cddis.2015.328)
Supplement: Supplementary Information [file cddis2015328x3.doc]

**Supplementary table 1. Correlation of HDAC1 and p21^WAF1^ expression and clinicopathological parameters in lung cancer patients．**

| **Variable** | **N** | **HDAC1 expression (%)** | ***P*-value** | **p21^WAF1^ expression (%)** | ***P*-value** |
| --- | --- | --- | --- | --- | --- |
| **Age** |  |  |  |  |  |
| < 65 years | 24 | 14(43.8%) | 0.60 | 8(38.1%) | 0.76 |
| ≥ 65 years | 35 | 18(56.2%) |  | 13(61.9%) |  |
| **Gender** |  |  |  |  |  |
| Male | 38 | 20 (62.5%) | 0.74 | 15(71.4%) | 0.40 |
| Female | 21 | 12(37.5%) |  | 6(28.6%) |  |
| **Histologic type** |  |  |  |  |  |
| Squamous carcinoma | 12 | 8(25%) | 0.33 | 5(23.8%) | 0.89 |
| Adenocarcinoma | 47 | 24(75%) |  | 16(76.2%) |  |
| **pTNM staging** |  |  |  |  |  |
| IIIb | 26 | 11(34.4%) | 0.10 | 12(57.1%) | 0.13 |
| IV | 33 | 21(65.6%) |  | 9(42.9%) |  |
