# Supplementary material for: Targeting HDAC with a novel inhibitor effectively reverses paclitaxel resistance in non-small cell lung cancer via multiple mechanisms
Source: Cell Death Dis. 2016 Jan 21;7(1):e2063–. doi: 10.1038/cddis.2015.328 (PMC4816165; doi:10.1038/cddis.2015.328)
Supplement: Supplementary Figure Legend [file cddis2015328x4.doc]

**Supplementary Figure 1 The effects of SNOH-3 on the expression of VEGF and KLF4 in HUVECs.** The proteins were assessed by western blot analyses in HUVEC cells after SNOH-3 (0.1, 0.8 and 5 μM) treated for 24h. β-actin expression was used as a loading control.
